# Echocardiographic assessment of diastolic dysfunction in elderly patients with severe aortic stenosis before and after aortic valve replacement

**DOI:** 10.1186/s12947-021-00262-1

**Published:** 2021-09-28

**Authors:** Hatice Akay Caglayan, Didrik Kjønås, Siri Malm, Henrik Schirmer, Assami Rösner

**Affiliations:** 1 Department of Cardiology, Division of Cardiothoracic and Respiratory Medicine, University Hospital of North Norway, 9038 Tromsø, Norway; 2 Institute of Clinical Medicine, The Arctic University of Norway, University of Tromsø (UiT), 9037 Tromsø, Norway; 3 Department of Gastrointestinal Surgery, University Hospital of North Norway, Tromsø, Norway; 4 Department of Internal Medicine, University Hospital of North Norway, Harstad, Norway; 5 Department of Cardiology, Akershus University Hospital, Lørenskog, Norway; 6 Institute of Clinical Medicine, Cardiovascular Research Group, Campus Ahus, University of Oslo, Oslo, Norway

**Keywords:** Left ventricular filling pressures, Aortic stenosis, Aortic valve replacement, Doppler echocardiography, Brain natriuretic peptide

## Abstract

**Background:**

The 2016 guidelines of the American Society of Echocardiography (ASE) and European Association of Cardiovascular Imaging (EACVI) for evaluation of left ventricular (LV) diastolic dysfunction by Doppler flow and tissue Doppler- echocardiography do not adjust assessment of high filling pressures for patients with aortic stenosis (AS). However, most of the studies on this patient group indicate age independent specific diastolic features in AS. The aim of this study is to identify disease-specific range and distribution of diastolic functional parameters and their ability to identify high N-terminal prohormone of brain natriuretic peptide (NT-proBNP) levels as a marker for high filling pressures.

**Methods:**

In this study, 169 patients who underwent surgical aortic valve replacement (SAVR) or transcatheter aortic valve replacement (TAVR) were prospectively enrolled. Resting echocardiography was performed including Doppler of the mitral inflow, pulmonary venous flow, tricuspid regurgitant flow and tissue Doppler in the mitral ring and indexed volume-estimates of the left atrium (LAVI). Echocardiography, and NT-proBNP levels were assessed before TAVR/SAVR and at two postoperative visits at 6 and 12 months.

**Results:**

Pre- and postoperative values were septal e′; 5.1 ± 3.9, 5.2 ± 1.6 cm/s; lateral e′ 6.3 ± 2.1; 7.7 ± 2.7 cm/s; E/e′19 ± 8; 16 ± 7 cm/s; E velocity 96 ± 32; 95 ± 32 cm/s; LAVI 39 ± 8; 36 ± 8 ml/m^2^, pulmonary artery pressure (PAP) 39 ± 8; 36 ± 8 mmHg, respectively. The scoring recommended by ASE/EACVI detected elevated NT pro-BNP with a specificity of 25%. Adjusting thresholds towards PAP ≥ 40 mmHg, E velocity ≥ 100 cm/s, E deceleration time < 220 ms, and E/septal e′ ≥ 20 or septal e′ < 5.0 cm/s increased prediction of NT-proBNP levels ≥500 ng/L with substantially improved specificity (> 85%).

**Conclusion:**

Diastolic echocardiographic parameters in AS indicate persistent impaired relaxation and NT-proBNP indicate elevated filling pressures in most of the patients, improving only modestly 6–12 months after TAVR and SAVR. Applying the 2016 ASE/EACVI recommendations for detection of elevated filling pressures to patients with AS, elevated NT pro-BNP levels could not be reliably detected. However, adjusting thresholds of the echocardiographic parameters increased specificities to useful diagnostic levels.

**Trial registration:**

The study was prospectively approved by the regional ethical committee, REK North with the registration number: REK 2010/397-10.

**Supplementary Information:**

The online version contains supplementary material available at 10.1186/s12947-021-00262-1.

## Introduction

Degenerative aortic valve stenosis (AS) is a chronic and progressive disease which gradually provokes afterload and thereby causes pressure overload on the left ventricle (LV), leading to ventricular fibrosis and consequently diastolic dysfunction over time [1–3]. The associated diastolic properties start with delayed relaxation at normal filling pressures, consecutively facilitating a state with increased filling pressures, and finally inducing the clinical image of heart failure with pulmonary congestion [4].

According to the 2016 recommendations of the American Society of Echocardiography (ASE) and the European Association of Cardiovascular Imaging (EACVI), elevated LV filling pressures should be assessed by left atrial volume index (LAVI) and blood-flow and tissue Doppler parameters, i.e. septal and/or lateral e′ average, E′\e′ ratio, peak gradient over the tricuspid regurgitation (TR_peak_) [4]. The ASE/EACVI recommend modified assessment-criteria for hearts with reduced EF and for hearts with special forms of cardiomyopathies.

However, in AS, they do not recommend modification of these criteria [4], even though altered relaxation and filling properties in AS are well known [5–10]. The elderly population with AS have a complex structure of ventricular diastolic properties with delayed relaxation related to age, hypertension and hypertrophy. Using unmodified criteria for AS might probably lead to over- or under-estimation of filling pressures. Consequently, the assessment of diastolic dysfunction in this population remains challenging [5–10].

During progression of diastolic dysfunction, LV wall stress, pressure and volume overload induce the secretion of BNP/ NT-proBNP (N-terminal prohormone of brain natriuretic peptide), a cardiac neurohormone which is secreted by cardiomyocytes in the ventricles. Several studies have shown that high plasma BNP/NT-proBNP levels correlate well with elevated filling pressures and severity of heart failure in patients with AS [11–13]. An age dependent increase of plasma BNP/NT-proBNP levels is observed in population-based studies, while recent studies indicate the association with LV hypertrophy or subclinical heart failure rather than age only [14, 15].

In this study, we aim to describe diastolic functional parameters in elderly patients with severe AS, at baseline and after aortic valve replacement (AVR). We also tested these parameters and their cut-off values to predict high NT-proBNP levels as a marker of elevated LV filling pressure.

## Methods

### Study population

In a prospective study between 2010 and 2013, 169 patients with severe symptomatic AS, who were eligible either for transcatheter AVR (TAVR) or surgical AVR (SAVR) at the University Hospital of North Norway Tromsø, were consecutively included in the study. The decision for TAVR or SAVR was made by a multidisciplinary cardiology team who determined the operation type (TAVR or SAVR) based on patients’ indications, technical feasibility, the risk for open heart-surgery, age, comorbidities and mental status. Patients who were unable to give informed consent, or with life expectancy of less than 12 months, or with low motivation for interventional treatment, were not offered aortic valve replacement of any kind. All 169 included participants in the study were invited to a pre-operative clinical assessment and echocardiography and NT-proBNP measurement and two repeated control investigations at 6 (± 1 month) and 12 (± 1 month) months after the operation.

### Patient demographics and clinical characteristics

Clinical characteristics, mortality and complications during and after surgery were obtained from the patients’ electronic journals. Patients were classified according to the global initiative for chronic obstructive lung disease (GOLD) classification for chronic obstructive pulmonary disease (COPD), and the patients with COPD of unknown grade were classified as having COPD grade 1. The patients who had a history of stroke or transient ischemic attacks, or significant (> 70%) stenosis of the carotid arteries, were classified as having cerebrovascular disease. Chronic and paroxysmal atrial fibrillation/flutter conditions were grouped as one variable. The patients with records, less than 2 weeks prior to surgery, of physician documented clinical signs of heart failure in the form of unusual dyspnoea on light exertion, orthopnoea, fluid retention, description of rales on auscultation, or pulmonary oedema on chest X-ray, were classified as having heart failure (HF < 2 weeks). The patients were classified as having left bundle branch block (LBBB) according to the Minnesota criteria in the resting ECG. Predominance of ventricular pacing or LBBB was assessed by the rhythm registered during echocardiography.

### Echocardiography

All patients underwent preoperative echocardiography in the left lateral decubital position with an iE33 scanner (S5–1 probe, Philips Medical systems, Andover, MA). Conventional 2-dimensional grey scale images were obtained in parasternal long- and short- axes as well as apical four-chamber, two-chamber and long-axis-views. 2D long-axis images were obtained at a time-resolution of was 58 ± 20 frames/s. LV EF was derived from the two- and four-chamber views using the biplane Simpson’s method [16]. The same two views were used to calculate LAVI at end-systole. The degree of AS was expressed using the mean gradient of the Doppler flow across the aortic valve and the indexed aortic valve area calculated using the continuity equation.

For the evaluation of mitral regurgitation and aortic regurgitation, we performed a multiparametric, semiquantitative approach as recommended in the guidelines [17].

Diastolic LV function was assessed by evaluation of the following parameters: Mitral E- and A-wave velocity, E/A ratio, E deceleration time (DT), septal, lateral wall and average tissue Doppler velocities (e′) and their E/e′ ratios, systolic filling fraction of the pulmonary veins (SFF), LAVI, the velocity and peak gradient over the tricuspid regurgitation (TR_peak_) and an estimate of the systolic pulmonary arterial pressure (PAP) by adding 10 mmHg to the TR_peak_, and the isovolumetric relaxation time (IVRT).

We applied the scoring system according to the ASE/EACVI 2016 guidelines based on at least two of the criteria for ventricles with normal EF: septal e′ ≤7 cm/sec and/or lateral e′ ≤ 10 cm/sec, average E/e′ ratio ≥ 14 cm/sec, TR_peak_ ≥ 2,8 m/sec and LAVI ≥34 ml/m2. We scored also relaxation properties based on the ASE/EACVI 2009 guidelines, which states the presence of impaired relaxation if septal e′ < 8; lateral e′ < 10 cm/s or LA ≥ 34 ml/m^2^.

Finally a new scoring system was established based on independent predictors of the multivariate-analysis, adding for each variable “− 1” for normal values, “0” for intermediate and + 1 for high probability of elevated filling pressures.

### Statistical analysis

Statistical analysis was performed using SPSS 25 and 26 (SPSS Inc., Chicago, IL, USA). Preoperative patient-characteristics of the TAVR and SAVR groups were compared using either t-test or chi-square test. The frequencies of categorized diastolic parameters were compared using chi-square test to analyse the differences between the age groups (≥ or < 80 years) and pre- and postoperative data baseline and control after TAVR/SAVR. We compared further pre-operative to post-operative echocardiographic diastolic parameters using paired t-test. For this comparison, one average value of each parameter was calculated for parameters of the first and second post-AVR control.

Receiver operating characteristic (ROC) curve analysis was performed on pre- and post-operative echocardiographic measurements when NT-proBNP values were available. The NT-proBNP level > 500 ng/L was chosen from the clinical cut-off value in our hospital for the largest age group of patients in our study (females and males 70–80 years). Age-related cut-off values were not chosen, since the NT-proBNP threshold increased in many patients during the first control-year. To identify predictors of elevated NT-proBNP levels, three cut-off values of similar sensitivity-specificity-sum were chosen for each diastolic parameter, among the values with high sensitivity and moderate specificity and high specificity combined with moderate sensitivity.

For the independent and dependent correlation of diastolic echocardiographic indices with the presence of NT-proBNP value > 500 ng/L, univariate and multivariate logistic regression analysis was performed. Variables with *p* ≤ 0.05 and deemed clinically relevant were selected and tested to analyse interaction and co-linearity prior to forward and backward multivariable logistic regression analysis. When the interaction terms in the backward analysis were non-significant, the forward model was used. For the final inclusion into a multiple regression model, a *p*-value of ≤0.05 was considered significant. Independent predictors of the multivariable analysis were combined in a scoring system. For this new score, sensitivities and specificities were calculated for different cut-off values using ROC curve analysis.

## Results

Over a three-year period, 169 patients were included in this study. Twelve patients with a mean gradient < 40 mmHg and reduced stroke-volume, indicating a low-flow low gradient AS, while two patients were referred to AVR and CABG presented with a moderate aortic stenosis. One patient referred to TAVR had a gradient of 38 mmHg and normal stroke volume. At the time of AVR referral, all patients presented with exertional dyspnoea at least NYHA II, 46% presented with angina, 6% with dizziness or syncope and 10% of patients presented with additional angina and dizziness.

There was no statistical difference for type of symptoms between the TAVI and AVR groups. Eighteen patients died during the procedure or during the first 6 months after SAVR or TAVR. One hundred thirty-five patients returned to either follow-up control, 132 participants attended the first postoperative control at 6 months, and 121 patients attended the second control at 12 months. Of 388 visits of the 169 included patients with NT-proBNP measurements, NT-proBNP values could be linked to echocardiographic parameters in 358 visits.

We compiled baseline demographic and clinical characteristics of the patients who underwent TAVR and SAVR (Table 1). The TAVR group consisted of 98 (49% male) patients and the SAVR group of 71 (56% male) patients. Patients in the TAVR group were significantly older, and had significantly higher body mass index (BMI), pulmonary artery pressure (PAP) and NT-proBNP levels than the patients in the SAVR group. More COPD, peripheral vascular disease (PVD) and heart failure < 2 weeks cases were observed in the TAVR than in the SAVR group. In line with renal dysfunction, pre- glomerular filtration rate (GFR) and post-GFR levels were significantly lower in the TAVR group than in the SAVR group. The prevalence of previous coronary artery bypass grafting (CABG) and percutaneous coronary intervention (PCI), Logarithmic European System for Cardiac Operative Risk Evaluation (LogEuroScore) and pre-operative functional New York Heart Association (NYHA) class III-IV, were significantly higher in the TAVR group, whereas the prevalence of new revascularization was significantly higher in the SAVR group. There were no statistically significant differences in terms of EF, hypertension, diabetes, plasma cholesterol and creatine kinase-MB (CK-MB) levels, smoking, coronary artery disease (CAD), cerebrovascular disease, and prevalence of LBBB and post-operative functional NYHA class III-IV between the two groups.

**Table 1:** Patient characteristics

	TAVR	SAVR	*P*-value
	n (%) or mean ± SD	n (%) or mean ± SD	
n	98	71	
Male	48 (49)	40 (56)	0.345
Age (y)	83 ± 5	78 ± 5	**< 0.0001**
BMI (kg/m^2^)	26 ± 5	27 ± 4	**0.018**
EF (%)	52 ± 13	55 ± 12	0.122
COPD	34 (35)	14 (20)	**0.030**
Cancer	20 (21)	15 (21)	0.480
Cerebrovascular disease	22 (22)	8 (11)	0.060
GFR pre (ml/min/1,73m^2^)	33 ± 12	38 ± 12	**0.002**
GFR post (ml/min/1,73m^2^)	35 ± 14	42 ± 17	**0.001**
PVD	32(33)	4 (6)	**0.001**
Hypertension	68 (70)	51 (72)	0.731
Diabetes	28 (29)	17 (24)	0.545
Smoking	13 (13)	8 (11)	0.698
Cholesterol (mmol/L)	4.7 ± 1.2	4.9 ± 1.1	0.322
PAP	40 ± 14	33 ± 10	**0.001**
CAD	69 (70)	42 (59)	0.323
Previous CABG	32 (33)	1 (1)	**< 0.0001**
New CABG	0 (0)	31 (44)	**< 0.0001**
Previous PCI	48 (49)	13 (18)	**0.002**
Pacemaker	13 (13)	5 (7)	0.252
LBBB	11 (11)	6 (9)	0.554
Heart failure < 2 weeks	80 (82)	43 (61)	**0.002**
CK MB post (U/L)	19 ± 58	28 ± 20	0.224
NT-proBNP (ng/L)	5416 ± 7920	1422 ± 2341	**< 0.0001**
NYHA III-IV pre	88 (89)	50 (70)	**0.002**
NYHA III-IV post	4 (6)	2 (3)	0.210
LogEuroScore	25 ± 13	10 ± 6	**< 0.0001**

*BMI* Body mass index, *CABG* Coronary artery bypass grafting, *CAD* Coronary artery disease, *CK* Creatine kinase, *COPD* Chronic obstructive pulmonary disease, *EF* Ejection fraction, *GFR* Glomerular filtration rate, *LBBB* Left bundle brunch blocks, *LogEuroScore* Logarithmic European System for Cardiac Operative Risk Evaluation, *NT-proBNP* N-terminal prohormone of brain natriuretic peptide, *NYHA* New York Heart Association, *PAP* Pulmonary artery pressure, *PCI* Percutaneous coronary intention, *PVD* Peripheral vascular disease, *SAVR* Surgical aortic valve replacement, *TAVR* Transcatheter aortic valve replacement

Comparing diastolic echocardiographic parameters and NT-proBNP levels in patients who were 80 years or older to patients younger than 80 years, we found no differences regarding mitral-inflow and tissue-Doppler parameters. We observed a borderline significantly higher LAVI in the older patients, while only NT-proBNP and PAP values in the older patients were significantly higher compared to those younger than 80 years.

**Fig. 1:**
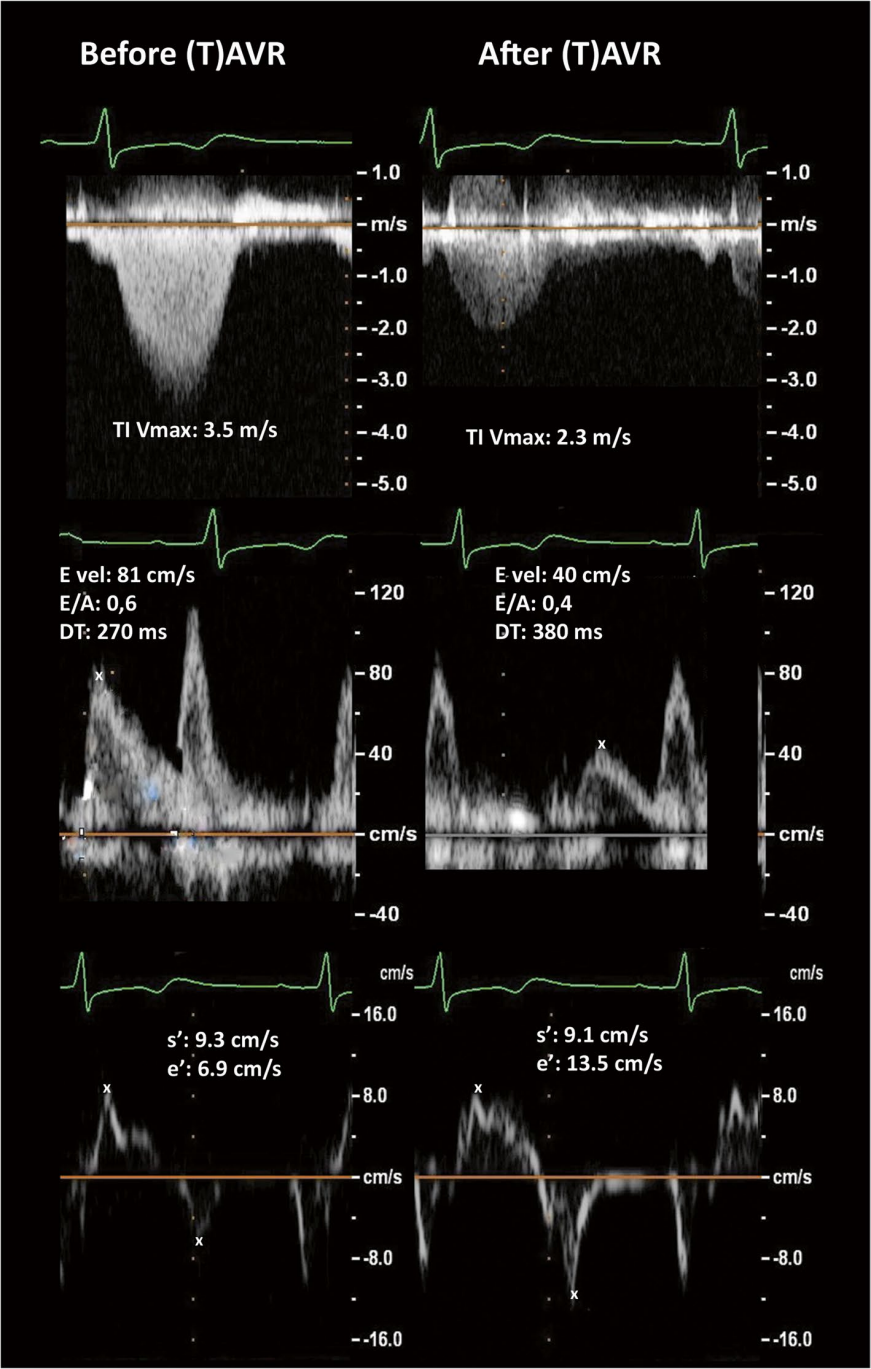
Example for mitral valve and tissue-Doppler parameters before and after aortic valve replacement (AVR); Upper panel: TI: tricuspid insufficiency; Vmax: maximal velocity; Mid panel: Mitral flow: E vel: E-wave velocity; E/A: ratio og E-wave and A-wave velocity; DT: deceleration time; Lower panel: Tissue Doppler: s’: peak systolic velocity; e’: peak e-velocity

Figure 1 shows a typical example for changes of mitral and tissue Doppler flow before and after TAVR/SAVR and Table 2 displays the comparison of diastolic parameters. For this analysis we calculated the mean-value between both follow-up visits. Paired t-test for all parameters comparing the first and second follow-up visit did not show any significant difference. As expected, most of the diastolic functional parameters improved after the TAVR/SAVR. In particular, NT-proBNP, PAP and PV SFF values were significantly decreased, and IVRT, lateral e′ and average e′, E DT and MV E/A values were significantly increased after the TAVR/SAVR procedure.

**Table 2:** Distribution of diastolic measurements in patients with severe aortic stenosis

		n	Range; percent within range (%)					Mean ± SD	*P* (Chi Square)
	Range (cm/s)		< 5	5–7	7–10	≥10			
e’septal (cm/s)	Before SAVR/TAVR	128	65%	24%	9%	2%		5.1 ± 3.9	0.166
	After SAVR/TAVR	132	53%	36%	10%	1%		5.2 ± 1.6	
e’lateral (cm/s)	Before SAVR/TAVR	128	31%	35%	29%	5%		6.3 ± 2.1	< 0.0001
	After SAVR/TAVR	132	12%	34%	38%	16%		7.7 ± 2.7	
e’avrg (cm/s)	Before SAVR/TAVR	128	44%	36%	18%	2%		5.7 ± 2.4	< 0.0001
	After SAVR/TAVR	132	21%	45%	33%	2%		6.5 ± 1.8	
	Range (cm/s)		< 8	8–12	12–16	16–20	≥20		
E/e’sept ()	Before SAVR/TAVR	125	3%	7%	18%	22%	50%	22 ± 11	0.222
	After SAVR/TAVR	130	3%	14%	24%	20%	39%	20 ± 10	
E/e’lat ()	Before SAVR/TAVR	128	7%	26%	25%	17%	25%	16 ± 8	0.051
	After SAVR/TAVR	132	13%	36%	24%	12%	14%	14 ± 7	
E/e’avrg ()	Before SAVR/TAVR	127	5%	12%	28%	26%	30%	19 ± 8	0.014
	After SAVR/TAVR	132	5%	28%	27%	23%	18%	16 ± 7	
	Range (ms)		< 60	60–80	80–100	100–120	> 120		
IVRT	Before SAVR/TAVR	128	40%	26%	17%	9%	7%	70 ± 44	0.007
	After SAVR/TAVR	123	24%	22%	21%	20%	13%	90 ± 54	
	Range (mmHg)		< 30	30–40	40–50	≥50			
PAP	Before SAVR/TAVR	122	17%	47%	22%	14%		39 ± 8	0.046
	After SAVR/TAVR	130	22%	55%	19%	5%		36 ± 8	
	Range (%)		< 25	25–40	50–60	50–80			
PV SFF	Before SAVR/TAVR	133	5%	17%	40%	38%		51 ± 17	0.048
	After SAVR/TAVR	133	9%	17%	50%	23%		49 ± 14	
	Range (ml/m^2^)		< 34	34–50	50–75	> 75			
LA volume Index	Before SAVR/TAVR	123	16%	33%	45%	7%		52 ± 20	0.093
	After SAVR/TAVR	129	16%	46%	33%	7%		50 ± 23	
	Range (cm/s)		< 50	50–80	80–110	> 110			
MV peak E	Before SAVR/TAVR	130	4%	28%	27%	42%		96 ± 32	0.415
	After SAVR/TAVR	132	3%	36%	21%	40%		95 ± 32	
MV peak A	Before SAVR/TAVR	102	10%	13%	26%	52%		102 ± 32	0.299
	After SAVR/TAVR	102	2%	22%	21%	56%		105 ± 28	
	Range ()		< 0.5	0.5–0.8	0.8–1.1	≥1.1			
MV E/A ()	Before SAVR/TAVR	101	13%	43%	27%	18%		1.0 ± 0.6	0.009
	After SAVR/TAVR	101	10%	41%	40%	11%		1.0 ± 0.4	
	Range (ms)		< 150	150–220	220–280	> 280			
E decel time	Before SAVR/TAVR	130	13%	30%	19%	39%		251 ± 95	0.050
	After SAVR/TAVR	132	5%	28%	30%	37%		260 ± 76	
	Range (ng/L)		< 500	500–850	850–1700	1700–5000	> 5000		
NT-proBNP	Before SAVR/TAVR	119	23%	16%	19%	30%	13%	3168 ± 6270	< 0.0001
	After SAVR/TAVR	120	43%	19%	21%	13%	4%	1274 ± 2238	
	Range ()		0 (Normal)	1 (Normal)	2 (inde-terminate)	3 (positive)	4 (positive)		
Score 2016	Before SAVR/TAVR	117	3%	9%	22%	40%	26%	2.7 ± 1.0	0.071
	After SAVR/TAVR	128	2%	13%	35%	36%	15%	2.5 ± 1.0	
	Range ()		0 (Normal)	1	2	3			
2009 Criteria for Grade I, II or III	Before SAVR/TAVR	127	1%	8%	20%	72%		2.6 ± 1	< 0.0001
	After SAVR/TAVR	130	2%	20%	28%	51%		2.3 ± 1	

2009 Criteria for Grade I, II or III diastolic dysfunction were septal e′ < 8, lateral e′ < 10, LA ≥34 ml/m2, displayed is the number of criteria met

*A* Late (atrial) diastolic transmitral flow velocity, *E* Early diastolic transmitral flow velocity, *e′* Early diastolic mitral annuler velocity, *IVRT* Isovolumic relaxation time, *LA* Left atrium, *MV dec time* Mirtal valve deceleration time, *MV peak E* Mitral valve early diastolic filling velocity, *MV peak A* Mitral valve late diastolic atrial filling velocity, *NT-proBNP* N-terminal prohormone of brain natriuretic peptide, *PAP* Pulmonary artery pressure, *PV SFF* Pulmonary veins systolic filling fraction, *SAVR* Surgical aortic valve replacement, *TAVR* Transcatheter aortic valve replacement

Using the 2016 ASE/EACVI recommended cut of values, we found 33 of 382 (8.6%) of measurements with PAP > 40 mmHg, 233 of 392 (59%) with E/e′ > 14 cm/s, 336 of 393 (85%) with LAVI> 34 ml/m^2^ and 346 of 392 (88%) with septal e′ < 7 cm/s. The NT-proBNP level was found to be ≥500 ng/L in 256 of 388 (66%) visits.

**Fig. 2:**
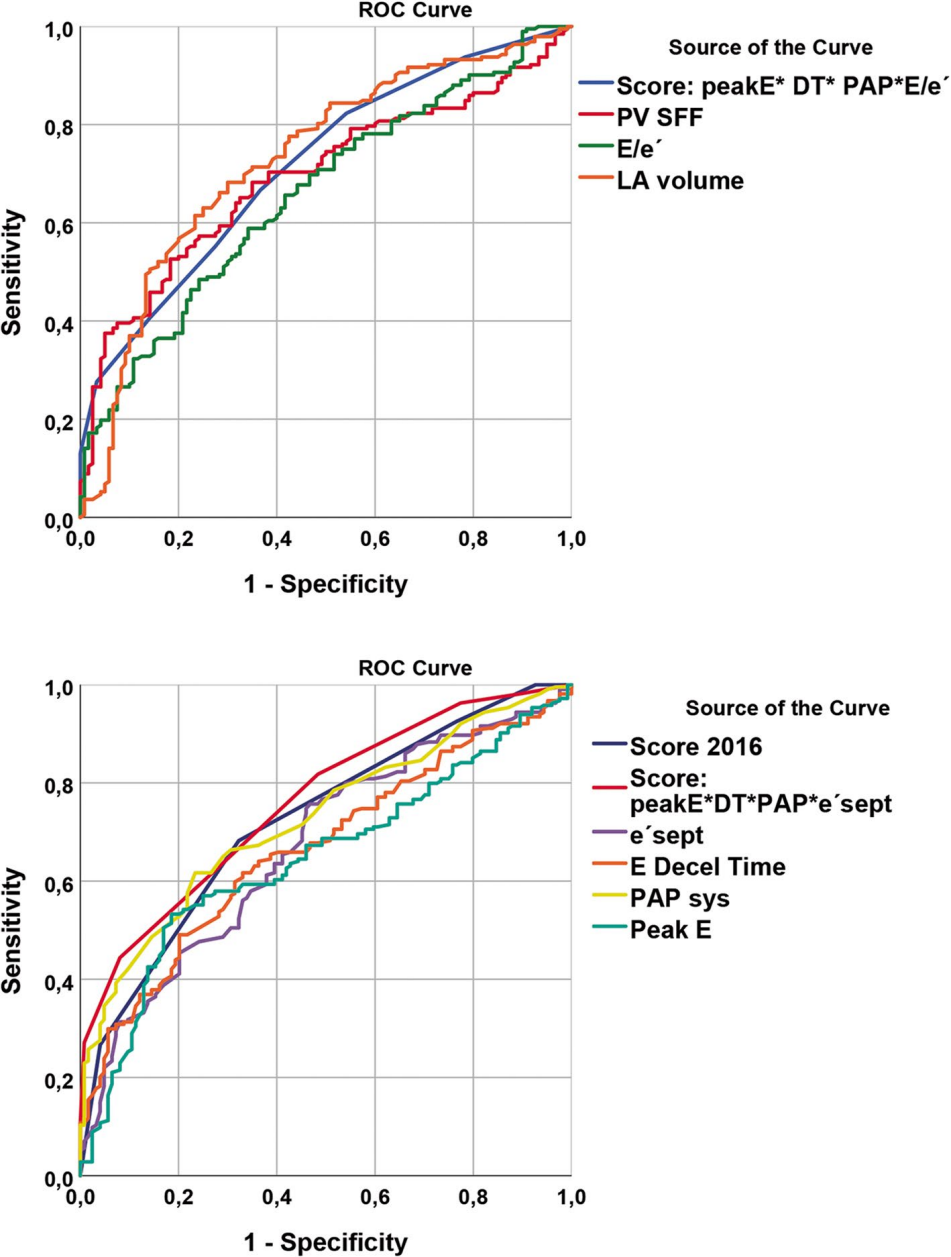
ROC curve analyses of echocardiographic diastolic parameters for NT-proBNP ≥500 ng/L. DT;deceleration time, E; early diastolic transmitral flow velocity, e′; early diastolic mitral annuler velocity, LA; left atrium, PAP; pulmonary artery pressure,, PV SFF; pulmonary veins systolic filling fraction, ROC; receiver operating characteristic

Figure 2 and Table 3 display the results of the ROC curve analysis of the ability of echocardiographic diastolic parameters to predict high NT-proBNP levels (≥ 500 ng/L). The same ROC curve analysis was also separately performed for pre- and post AVR visits. These tables are added as supplementary material, showing no significant difference between AUCs or sensitivity/specificity at the same cut-off values. Even though NT-proBNP after AVR was significantly reduced, the diastolic properties after afterload-reduction indicate persistent diastolic dysfunction.

**Table 3:** ROC curve analysis of echocardiographic parameters to predict NT-proBNP ≥500 ng/L in patients with severe aortic stenosis

*N* = 210 NT-proBNP ≥500 *N* = 123 NT-proBNP < 500	AUC	CI Lower bound	CI Upper bound	*P*-value
e’septal (cm/s)	0.68	0.62	0.73	**< 0.0001**
e’lateral (cm/s)	0.62	0.55	0.68	**0.001**
e’avrg (cm/s)	0.62	0.56	0.68	**0.002**
E/e’sept ()	0.70	0.65	0.76	**< 0.0001**
E/e’lat ()	0.63	0.56	0.69	**< 0.0001**
E/e’avrg ()	0.67	0.61	0.73	**< 0.0001**
PV SFF	0.68	0.63	0.74	**< 0.0001**
LA volume (ml)	0.73	0.67	0.79	**< 0.0001**
MV peak E (cm/s)	0.64	0.58	0.70	**0.050**
MV peak A (cm/s)	0.50	0.44	0.57	0.939
MV E/A ()	0.57	0.51	0.64	**0.037**
MV dec time (ms)	0.66	0.61	0.72	**< 0.0001**
IVRT (ms)	0.56	0.50	0.62	0.057
PAP (mmHg)	0.68	0.63	0.74	**< 0.0001**
Score 2016	0.72	0.67	0.78	**< 0.0001**
Score 1 e’sept	0.76	0.71	0.82	**< 0.0001**
Score 2 E/é avrg	0.70	0.65	0.77	**< 0.0001**

*A* Late (atrial) diastolic transmitral flow velocity, *AUC* Area Under the Curve, *CI* Confidence interval, *E* Early diastolic transmitral flow velocity, *e′* Early diastolic mitral annuler velocity, *IVRT* Isovolumic relaxation time, *LA* Left atrium, *MV dec time* Mirtal valve deceleration time, *MV peak E* Mitral valve early diastolic filling velocity, *MV peak A* Mitral valve late diastolic atrial filling velocity, *NT-proBNP* N-terminal prohormone of brain natriuretic peptide, *PAP* Pulmonary artery pressure, *ROC* Receiver operating characteristic

Table 4 shows the results of the univariable and multivariable regression analyses of the same echocardiographic diastolic parameters to determine high NT-proBNP levels. Septal and average e′, septal, lateral and average E/e′, PV SFF, LA volume, MV peak E, MV DT and PAP showed significant correlation with high NT-proBNP levels, whereas lateral e′, MV peak A, MV E/A and IVRT showed no significant correlation in univariable regression analysis. Multivariable regression analysis revealed septal e′ and E/e′, MV DT and PAP as independent significant markers of high NT-proBNP levels.

**Table 4:** Univariate and multivariate regression analysis for indicators of elevated NT-proBNP (≥500 ng/L)

	Univariate regression (Unadjusted model)				Multivariate regression (Adjusted model)			
	OR	95% CI	95%C I	*p*-value	OR	95% CI	95% CI	*p*-value
e’septal (cm/s)	0.731	0.632	0.845	< 0.0001	1.196	1.118	1.279	< 0.0001*
e’lateral (cm/s)	0.921	0.847	1.002	0.056				
e’avrg (cm/s)	0.807	0.713	0.913	0.001				
E/e’sept ()	1.108	1.071	1.147	< 0.0001	1.196	1.118	1.1279	< 0.0001*
E/e’lat ()	1.079	1.039	1.127	< 0.0001				
E/e’avrg ()	1.090	1.040	1.142	< 0.0001				
PV SFF	0.947	0.929	0.965	< 0.0001				
LA volume (ml)	1.022	1.013	1.030	< 0.0001				
MV peak E (cm/s)	1.019	1.010	1.027	< 0.0001	0.965	0.949	0.982	0.001
MV peak A (cm/s)	0.999	0.991	1.007	0.782				
MV E/A ()	2.278	1.210	4.289	0.011				
MV dec time (ms)	0.994	0.991	0.997	< 0.0001	0.994	0.991	0.998	0.001
IVRT (ms)	0.997	0.993	1.001	0.192				
PAP (mmHg)	1.114	1.097	1.192	< 0.0001	1.095	1.039	1.154	0.001

*E’sept or E/é avrg exclude each other in the equation and can be used interchangeably

*A* Late (atrial) diastolic transmitral flow velocity, *E* Early diastolic transmitral flow velocity, *e′* Early diastolic mitral annuler velocity, *IVRT* Isovolumic relaxation time, *LA* Left atrium, *MV dec time* Mirtal valve deceleration time, *MV peak E* Mitral valve early diastolic filling velocity, *MV peak A* Mitral valve late diastolic atrial filling velocity, *NT-proBNP* N-terminal prohormone of brain natriuretic peptide, *PAP* Pulmonary artery pressure, *PV SFF* Pulmonary veins systolic filling fraction

**Fig. 3:**
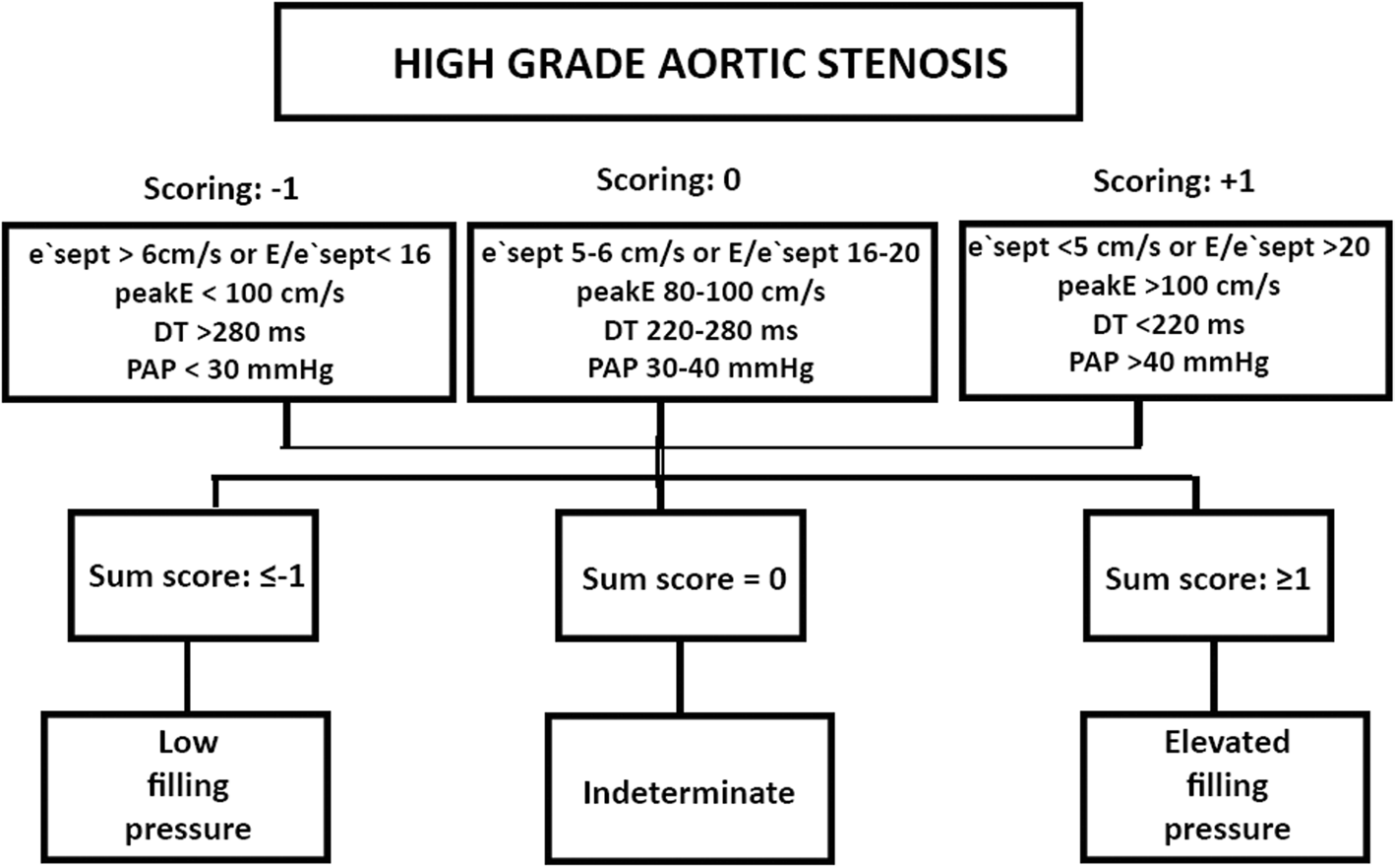
Scoring for elevated filling pressures based on the results of the present study

Table 5 displays cut-off values based on high sensitivity (left row), intermediate sensitivity and specificity (middle row) and high enough specificity to reliably indicate the presence of elevated filling pressure (right row). For the scoring the value − 1 was applied for negative scoring using cut-off A, 0 (indeterminate) for results between cut-off A and C and + 1 for positive scoring using cut-off C. For each of the four scoring-parameters, a value was applied and the sum of ≥1.0 indicated increased NT-proBNP, while a score of ≤ − 1 indicate normal NT-proBNP. The scoring-algorithm is displayed in Fig. 3. Specificity for the ASE/EACVI recommended algorithm was 23% in the AS population, while our suggested scorings with adjusted cut-off values displayed acceptable specificities (> 80%).

**Table 5:** Cut-off values, sensitivity and specificity for detection of elevated NT-proBNP

	N pos	N neg	A			B			C		
			Cut-off A	Sensitivity (%)	Specificity (%)	Cut-off B	Sensitivity (%)	Specificity (%)	Cut off C	Sensitivity (%)	Specificity (%)
e’septal (cm/s)	227	129	6.0	83	35	5.5	75	53	5.0	64	60
e’avrg (cm/s)	227	129	7.0	72	39	6.0	65	56	5.5	45	72
E/e’sept ()	227	129	16	72	50	18	64	65	20	55	76
E/e’avrg ()	227	129	14	67	57	15	58	66	16	54	70
PV SFF	220	128	60	80	41	55	67	63	50	55	77
LA volume (ml)	227	130	70	85	43	80	73	60	90	61	75
PAP (mmHg)	220	128	30	84	31	35	61	74	40	41	90
Peak E (cm/s)	228	130	80	68	45	90	59	63	100	52	81
E DT (ms)	228	130	280	68	52	250	59	68	220	44	81
Score of the parameters above			<Cut-off A: -1			Cut-off A to C = 0			>Cut-off C: +1		
Sum Score of e’sept*peakE* DT *PAP	219	125	−1.5	82	51	−0.5	62	73	0.5	45	92
Sum Score of: E/ e’sept *peakE *DT *PAP	196	123	−1.5	66	63	−0.5	55	72	0.5	40	86
Score 2016	214	124	1.5	93	23	2.5	68	68	3.5	27	96

Score: sum of score for the three independent parameters <cutoff A = -1; between cut-off A and C: 0; ≥cut-off C = 1

*DT* Deceleration time, *E* Early diastolic transmitral flow velocity, *e′* Early diastolic mitral annuler velocity, *IVRT* Isovolumic relaxation time, *LA* Left atrium, *NT-proBNP* N-terminal prohormone of brain natriuretic peptide, *PAP* Pulmonary artery pressure, *PV SFF* Pulmonary veins systolic filling fraction

## Discussion

This study demonstrates range and distribution of echocardiographic parameters in the elderly population with high grade aortic stenosis. Independent predictors of elevated NT-proBNP levels (> 500 ng/L) are PAP, mitral peak E, DT and septal E/e′ or septal e′ which can be used interchangeably. Parameters which are recommended for the assessment of diastolic dysfunction according to the 2016 ASE and EACVI guidelines (i.e. high average E/e′, low septal e velocity, high TR velocity and high LAVI) result in comparable AUC in ROC curve analyses as reported in the guidelines. However, specificities at sensitivity > 60%; > 45% could be increased from 23 to > 75%; 85%, respectively, by appropriate change of threshold-values.

### Diastolic dysfunction in aortic stenosis, hemodynamic considerations

Aortic stenosis modifies ventricular diastolic properties with three different mechanisms:

1. Delayed relaxation and relaxation velocity of the mitral ring might be associated with increased afterload and left ventricular hypertrophy [18]. Filling pressures can be normal in delayed relaxation [18]. Delayed relaxation velocity is expressed by reduced e′ in the septal or lateral ventricular wall. Low pressure gradient due to low ventricular pressure fall and prolonged early filling period are reflected by low E velocity, prolonged IVRT and prolonged DT [19].
2. Increased filling pressures can be determined by invasive measurement of ventricular pre-A pressure, which is a condition often followed by increased end-diastolic pressure and pulmonary capillary wedge pressure (PCWP). Increased filling pressures in AS are often reversible and can occur at superimposed afterload in a ventricle with reduced relaxation properties and initially normal filling pressures [18]. Filling pressures can typically occur at decreased stroke volume, shortened diastole during tachycardia or at missing compensatory atrial filling in atrial fibrillation. Increased diastolic pressure gradients are associated with higher E velocities, shortened DT and IVRT, reduced PV SFF and higher PAP [4, 19]. Additionally, LA size and E/é are associated with increased PCWP at rest and during exercise [20].
3. Ventricular hypertrophy, fibrosis and molecular mechanisms of diastolic dysfunction [21] might affect ventricular relaxation, and might also increase ventricular stiffness. These conditions, in turn, increase LV filling pressures with a non-reversible component after afterload reduction, and might contribute to persistently elevated NT-proBNP levels after TAVR/SAVR.

### Impaired relaxation in elderly AS patients

Most echocardiographic parameters of diastolic dysfunction have a U-shaped function with normal values between two extremes of either impaired relaxation or increased filling pressures [19]. For correct interpretation of indicators on increased filling pressures, diastolic properties of the LV at normal filling pressures in AS need to be considered. Results of our study and previous investigations [5, 20, 22] on diastolic parameters in AS, indicate that most AS LVs predominantly display signs of impaired relaxation. This is reflected by low e′, which increases after TAVR/SAVR, though without complete normalization [10, 23].

Age alone is one important factor for reduced relaxation properties. However, the patient population with TAVR, typically ages > 75 years, is scarcely represented in epidemiological studies [24] or in control groups of clinical studies. Probably due to the low number of study subjects, the 2016 ASE and EACVI guidelines refer to the high age group as > 60 years [4].

Compared to the high age group of the guidelines, the septal and lateral e′ are significantly lower, and LAVI and DT are significantly higher in the present study. To a smaller extent, age might reduce relaxation properties in higher age groups, however, most of the diastolic parameters were not different between the two age groups ≥80 and < 80 years in the present study.

Steine et al. [5] investigated patients (65 ± 12 years) with moderate AS, showing decreased septal e′ and increased atrial velocity compared to age-matched controls. Furthermore, E velocity, DT and E/e′ ratio were highly elevated compared to age-matched controls (17,4 ± 10 vs 11 ± 4) [5]. These results demonstrate that AS has age independent effects on ventricular diastolic properties.

Even though the present study represents an older patient population with higher degree of AS compared to Steines and other younger study populations [5, 10, 23, 25], we found a similar reduction of e′. Our results stress the predominance of factors other than age like LV hypertrophy, changed LV stiffness and increased afterload as the main reasons for decreased relaxation properties in AS patients. PAP was the only diastolic echocardiographic parameter which was significantly different between age-groups. One explanation of this could be low LV compliance with elevated filling pressures due to diffuse myocardial fibrosis in the elderly patients. Another explanation might be the high number of COPD patients in the elderly TAVR group.

### Changes of diastolic properties after TAVR/SAVR

Even though e′ increases after TAVR/SAVR [10, 23, 25–27], average e′ stayed lower than 10 cm/s in 90% of postoperative AS patients, indicating persistent impaired relaxation also after afterload reduction with AVR. Postoperatively, relaxation velocities in the lateral but not septal wall were significantly higher than pre-operative values, suggesting regionally inhomogeneous response to reduced afterload.

The correlation of e′ and improvement of s′ and their increase after AVR, indicate an association between ventricular contraction and relaxation velocities, which are both influenced by afterload changes [18, 28]. These findings point to afterload dependent, reversible reduction of contraction and relaxation velocities of the lateral wall, whereas the bulky, structurally changed septum of hypertensive ventricles [29] have less ability to recover. The majority of previous studies report a similar degree of diastolic dysfunction in AS patients with reduced recovery of relaxation properties [10, 23, 25–27], while only one study could be identified describing normalized E/e′, e′ and LA volume post TAVR [2]. It can be assumed that 1 year follow up might mainly reflect reduction of filling-pressures as shown by substantially lowered NT-pro BNP, while relaxation properties might be more dependent on a slower process of re-remodeling. If impaired relaxation is fully reversible, has to be shown in future long-term follow-up studies.

Kjønås et al. investigated echocardiographic systolic and diastolic parameters as predictors of mortality in the TAVR population of this study. These data showed that only increased pulmonary artery pressure was a predictor for early death, while other echocardiographic parameters of systolic or diastolic function did not indicate the outcome during the first 2 years.

### Indicators for increased filling pressures

The present study refers to the 2016 ASE/EACVI recommendations for the evaluation of LV diastolic dysfunction [4], which propose a simplified approach of estimating increased filling pressures (i.e. grade II and grade III of diastolic dysfunction). For assessment of impaired relaxation (i.e. Grade I diastolic dysfunction) we refer to the better definition of this state in the recommendations of 2009 [19].

The current results reflect the difficulties of estimating filling pressures in ventricles with highly impaired relaxation. Impaired relaxation will naturally change thresholds for increased filling pressures due to the U-shaped function of many Doppler-based parameters. This applies for mitral peak E velocity, DT, IVRT and PV SFF [12]. In opposite, the parameters e′ increases and E/e′ decreases with an additive effect of both, impaired relaxation and increased filling pressures. In AS patients, E/e′ seem to be predominantly influenced by ventricular relaxation properties (i.e. e′) rather than mitral pressure gradients (i.e. E velocity). Thus, e′ is lower in AS patients, while E-wave velocities are not elevated compared to age-matched controls [4, 5]. Similarly, comparing pre- and post AVR parameters shows that reduction of E/e′ was mainly related to postoperatively increasing e′, while E velocity was unchanged.

### NT-proBNP

Plasma NT-proBNP levels correlate well with elevated filling pressures in many different settings [1, 11, 13]. For this reason, in the present study, NT-proBNP was chosen as a marker for increased end-diastolic pressures. The cut-off NT-proBNP value of 500 ng/L was derived from the clinical normalcy range for females between 70 and 80 years and males ≥70 years, appropriate for the majority of our patients.

Significantly reduced NT-proBNP levels after AVR indicate moderately improved but not normalized filling pressures following afterload reduction. ROC curve analyses for several diastolic parameters distinguish high and low NT-proBNP values with AUC values between 65 and 76%, which indicates moderate diagnostic value when values are measured in the intermediate range. In line with our findings, Sasaki et al. showed that E/e′ was a highly sensitive and specific predictor of NT-proBNP levels, even after adjustment for clinical and systolic parameters [14]. Notably, in the present study, E/e′ was not superior to low septal e′ as a marker for high NT-proBNP levels. Even though high E/é is a widely used marker for elevated filling pressures [1, 11, 14], changes in filling are thought to be driven by changing peak E velocity, while low e′ is thought to be closer related to impaired relaxation [19]. However, the close correlation of e′ to NT-proBNP indicate that afterload and filling pressures have a direct influence on e′. A previous experimental study [18], tried to explain this inverse correlation by showing partially irreversible relation of impaired relaxation to increased filling pressures. Interestingly, peak E velocity showed low correlation with E/e′ or e′, and appeared to be an independent predictor of high NT-proBNP.

High PAP is a consistent and independent marker of elevated filling pressures [1, 4, 14] and a predictor for outcomes after TAVR and SAVR [25, 30]. In accordance with previous findings, high PAP values showed independent correlation with elevated NT-proBNP levels [1]. In consistency with the 2016 guidelines, this study shows that PAP is the most specific prognosticator with incremental effect to other parameters for detection of high filling pressures.

### Clinical implication

Preoperatively and postoperatively, 91 and 78% of patients with AS, respectively, displayed at least 2–3 of 3 criteria of grade I-III diastolic dysfunction, which include septal e′, lateral e′, LAVI, according to the 2009 ASE/EACVI guidelines.

Following the 2016 guidelines, all mitral flow and tissue-Doppler based parameters indicated elevated filling pressures in the majority of AS patients with unacceptably low specificity (25%). Only TR velocity > 2.8 m/s indicated high NT-proBNP levels with high specificity at acceptable sensitivity.

According to the results of the present study, we suggest the use of an adjusted model for elderly patients with AS by taking the following considerations into account:

First, E/e′ and e’are good indicators of elevated filling pressures. Because of high correlation with each other, they have no significant additive value and can be used interchangeably. Septal or average E/e′ and e′ seemed to be more accurate than lateral e′ measurements in assessment of increased filling pressure.

Second, LA volume was not a significant indicator of increased NT-proBNP levels and thus of lesser value in the assessment of increased filling pressures in AS patients. LA size changes with atrial fibrillation, which was present in 25% of the AS patients, and it increased due to impaired relaxation which is present in the majority of AS patients. LA size might be a better indicator for long-term increased filling pressures when seperatedly assessed for patients with sinus-rhythm or atrial fibrillation.

Third, our study confirms a highly specific PAP cut-off level of 40 mmHg which is the equivalent of TR peak velocity of 2.8 m/s suggested in the 2016 guidelines.

Fourth, E wave velocity and E DT, are independent indicators of elevated NT-proBNP levels in the multiple logistic regression analysis. E wave velocity decreases and DT is prolonged after AVR, indicating a closer relationship with reversible pressures. However, E velocity, DT, E/e′ and e′ cut-off values have to be adjusted towards cut-off values with higher specificities to be relevant for clinical use. According to our results, at least one of the following parameters need to cross their thresholds to indicate high filling pressures in patients with pre- or postoperative aortic stenosis: E/e’sept > 20; E velocity > 100 cm/s; DT < 220 ms; PAP > 40 mmHg or e’sept < 5.0 cm/s.

### Limitation of the study

The gold standard for diastolic filling pressures is invasive pressure measurements by right or left-heart catheter, which were not available in the present study. We used NT-proBNP as a surrogate marker of increased filling pressure, as previously practiced in other studies [17, 19]. Even though NT-proBNP correlates well with diastolic pressures, it is not known whether age alone increases proBNP. Setting the cut-off value of NT-proBNP at 500 ng/L did not take age-adjusted normalcy into account. However, a model with age-adjusted cut-off values for NT-proBNP was tested and rendered similar results.

The present study was performed on one ultrasound-system and the same reader, resulting in highly robust tissue velocity measurements. However, tissue Doppler indices are known to differ between ultrasound-systems with vendor specific machine-settings [31]. Comparison of E/e′ and e′ and cut-off values with guidelines or other studies are therefore challenging as varying results might be due to systematic errors.

In the TAVR population of this study, we could show that pulmonary hypertension is a strong predictor for early death [30]. Early drop-out of the patients with pulmonary hypertension might have influenced the postoperative echocardiographic measurements. However, the supplementary tables S1 and S2 indicate that sensitivity and specificity for elevated NT-proBNP pre- and post AVR is similar during the first year.

Many factors like valvular heart disease, atrial fibrillation, ejection fraction, age, gender, ischemic heart disease, hypertension, amyloidosis, mitral ring calcification [32] and others are known to influence diastolic properties of the heart. Unfortunately, the study population was too small to correct our results for all these factors. Larger studies based on echocardiography need to be conducted in order to take all these factors into account.

## Conclusion

Diastolic echocardiographic parameters in AS indicate persistent impaired relaxation and NT-proBNP indicate higher filling pressures in most of the patients, improving only modestly 6–12 months after TAVR and SAVR. Applying the 2016 ASE/EACVI recommendations for detection of elevated filling pressures to patients with AS, elevated NT pro-BNP levels could not be reliably detected. However, adjusting thresholds of the echocardiographic parameters increased specificities to useful diagnostic levels.

## Supplementary Information

**Additional file 1: Table S1.** ROC curve analysis of echocardiographic parameters to predict NT-proBNP ≥500 ng/L in patients with severe aortic stenosis. **Table S2.** Cut-off values, sensitivity and specificity for detection of elevated NT-proBNP.

## Data Availability

The datasets used and/or analysed during the current study are available from the corresponding author on reasonable request.

## Abbreviations

AS: Aortic stenosis
ASE: American Society of Echocardiography
AVR: Aortic valve replacement
AUC: Area under the curve
DT: Deceleration time
e′: Peak tissue velocity in early diastole
EACVI: European Association of Cardiovascular Imaging
IVRT: Isovolumetric relaxation time
LAVI: Left atrial volume index
LBBB: Left bundle branch block
LV: Left ventricle/left ventricular
NT-proBNP: N-terminal prohormone of brain natriuretic peptide
PAP: Pulmonary arterial pressure
PV SFF: Systolic filling fraction of the pulmonary veins
ROC: Receiver operator characteristics
SAVR: Surgical aortic valve replacement
TAVR: Transcatheter aortic valve replacement
TR: Tricuspid regurgitation
