# Supplementary material for: Echocardiographic assessment of diastolic dysfunction in elderly patients with severe aortic stenosis before and after aortic valve replacement
Source: Cardiovasc Ultrasound. 2021 Sep 28;19:32. doi: 10.1186/s12947-021-00262-1 (PMC8480046; doi:10.1186/s12947-021-00262-1)
Supplement: Supplementary file 1 — Additional file 1: Table S1. ROC curve analysis of echocardiographic parameters to predict NT-proBNP ≥500 ng/L in patients with severe aortic stenosis. Table S2. Cut-off values, sensitivity and specificity for detection of elevated NT-proBNP. [file 12947_2021_262_MOESM1_ESM.docx]

Supplement:

| Table S1: ROC curve analysis of echocardiographic parameters to predict NT-proBNP ≥ 500 ng/L in patients with severe aortic stenosis | | | | |
| --- | --- | --- | --- | --- |
| Before AVR  N= 89 NT-proBNP ≥ 500  N= 26 NT-proBNP < 500  After AVR  N= 100 NT-proBNP ≥ 500  N= 130 NT-proBNP < 500 | AUC | CI  Lower bound | CI  Upper bound | *p*-value |
| e`septal (cm/s) before | 0.68 | 0.56 | 0.81 | **0.005** |
| e`septal (cm/s) after | 0.64 | 0.57 | 0.72 | **<0.0001** |
| E/e`sept () before | 0.74 | 0.63 | 0.85 | **0.014** |
| E/e`sept () after | 0.68 | 0.61 | 0.75 | **<0.0001** |
| MV peak E (cm/s) before | 0.66 | 0.55 | 0.77 | **<0.0001** |
| MV peak E (cm/s) after | 0.64 | 0.57 | 0.71 | **<0.0001** |
| MV dec time (ms) before | 0.72 | 0.61 | 0.83 | **0.001** |
| MV dec time (ms) after | 0.65 | 0.58 | 0.72 | **<0.0001** |
| PAP (mmHg) before | 0.69 | 0.59 | 0.80 | 0.003 |
| PAP (mmHg) after | 0.71 | 0.64 | 0.78 | **<0.0001** |
|  |  |  |  |  |
| AUC; Area Under the Curve, CI; confidence interval, E; early diastolic transmitral flow velocity, e`; early diastolic mitral annuler velocity, MV dec time; mirtal valve deceleration time, MV peak E; mitral valve early diastolic filling velocity; NT-proBNP: N-terminal prohormone of brain natriuretic peptide, PAP; pulmonary artery pressure, ROC; receiver operating characteristic. | | | | |

| Table S2: Cut-off values, sensitivity and specificity for detection of elevated NT-proBNP | | | | | | | | | | | |
| --- | --- | --- | --- | --- | --- | --- | --- | --- | --- | --- | --- |
|  |  |  | A | | | B | | | C | | |
|  | N pos | N neg | Cut-off A | Sensitivity (%) | Specificity (%) | Cut-off B | Sensitivity (%) | Specificity (%) | Cut off C | Sensitivity (%) | Specificity  (%) |
| e`septal (cm/s) before | 89 | 26 | 6.0 | 87 | 38 | 5.5 | 78 | 50 | 5.0 | 63 | 58 |
| e`septal (cm/s) after | 130 | 100 | 6.0 | 79 | 35 | 5.5 | 72 | 55 | 5.0 | 52 | 68 |
| E/e`sept () before | 89 | 26 | 16 | 81 | 46 | 18 | 75 | 54 | 20 | 64 | 73 |
| E/e`sept () after | 130 | 100 | 16 | 67 | 50 | 18 | 58 | 66 | 20 | 51 | 76 |
| Peak E (cm/s) before | 89 | 26 | 80 | 73 | 42 | 90 | 58 | 65 | 100 | 55 | 85 |
| Peak E (cm/s) after | 130 | 100 | 80 | 66 | 47 | 90 | 60 | 61 | 100 | 53 | 79 |
| E DT (ms) before | 89 | 26 | 280 | 67 | 69 | 250 | 55 | 77 | 220 | 43 | 85 |
| E DT (ms) after | 130 | 100 | 280 | 68 | 50 | 250 | 60 | 67 | 220 | 42 | 81 |
| PAP (mmHg) before | 89 | 26 | 30 | 87 | 31 | 35 | 45 | 79 | 40 | 35 | 99 |
| PAP (mmHg) after | 130 | 100 | 30 | 82 | 31 | 35 | 55 | 79 | 40 | 32 | 94 |
| E; early diastolic transmitral flow velocity, e`; early diastolic mitral annuler velocity, MV dec time; mirtal valve deceleration time, MV peak E; mitral valve early diastolic filling velocity; NT-proBNP: N-terminal prohormone of brain natriuretic peptide, PAP; pulmonary artery pressure, ROC; receiver operating characteristic. | | | | | | | | | | | |
